# Identification of suitable reference genes for mesenchymal stem cells from menstrual blood of women with endometriosis

**DOI:** 10.1038/s41598-021-84884-5

**Published:** 2021-03-08

**Authors:** Victoria S. Zucherato, Leticia B. C. Penariol, Lilian E. C. M. Silva, Cristiana C. Padovan, Omero B. Poli-Neto, Julio C. Rosa-e-Silva, Rui A. Ferriani, Juliana Meola

**Affiliations:** 1 Gynecology and Obstetrics Department, Ribeirao Preto Medical School, University of Sao Paulo, Bandeirantes Avenue, 3900, 8th Floor of the Clinic Hospital, Universitary Campus, Ribeirao Preto, SP 14049-900 Brazil; 2 Multiuser Laboratory, Ribeirao Preto Medical School, University of Sao Paulo, Ribeirao Preto, SP Brazil; 3 National Institute of Hormones and Woman’s Health, CNPq, Porto Alegre, RS Brazil

**Keywords:** Molecular biology, Mesenchymal stem cells, Endocrine reproductive disorders, Infertility

## Abstract

It has been suggested that menstrual blood-derived mesenchymal stem/stromal cells (MenMSCs) are associated with the etiopathogenesis of endometriosis and considerable effort has been invested in searching for target genes and deciphering associated molecular pathways. However, reference gene stability for proper reproducible normalization in the analyses of the expression data validation is still unexplored in this experimental context. Therefore, in this exploratory study, we used stringent case and control selection criteria and collected menstrual blood from women with a laparoscopic diagnosis of advanced endometriosis and from fertile women without endometriosis. We tested for the first time the stability of 32 candidate reference genes to achieve increased accuracy and reliable results in the quantification of gene expression and direct future experiments using reverse transcription-quantitative PCR (RT-qPCR) in MenMSCs for endometriosis studies. Using the RefFinder web tool, we recommend the *EIF2B1* and *POP4* reference genes for the normalization of RT-qPCR data in study designs similar to ours. Furthermore, we suggest avoiding the commonly used *GAPDH* and *ACTB* reference genes as they are unstable. This high-visibility study is capable of directing different experimental designs as MenMSCs are derived from a minimally invasive tissue source with multifunctional roles in regenerative medicine.

## Introduction

Endometriosis is a benign estrogen-dependent gynecologic disease characterized by endometrium-like tissue outside of the uterine cavity. It is estimated that over 176 million women are affected by this condition globally, with women of reproductive age being more frequently affected^1,2^. The most widely accepted explanation for the etiology of endometriosis is Sampson’s theory, which considers the retrograde menstrual flow of endometrial tissue to the peritoneal cavity through the uterine tubes^3^. Although this theory is plausible, it does not explain all the nuances of the disease. Recently, it has been found that the endometrium, endometrial lesions and menstrual flow present cells with progenitor potential [known as endometrial mesenchymal stem/stromal cells (eMSCs) when obtained from the endometrium and lesions, and menstrual blood-derived stem/stromal cells (MenMSCs) when derived from menstrual flow] that might be directly related to the genesis of the disease^4^.

In parallel with the discovery of these cells, high-throughput methodologies have rapidly developed, enabling great advances in the understanding of numerous diseases. One of these advances concerns the gene expression analysis of the eutopic and ectopic endometrium, which has supported the identification of pathways and molecular processes involved in the physiopathology of endometriosis^5–7^. Although these methods are reliable, they have some constraints. One critical issue is the validation of identified genes. The gold standard method for this validation is reverse transcription-quantitative PCR (RT-qPCR)^8^.

RT-qPCR is a robust, reproducible, highly sensitive, and specific method with rapid detection that is capable of identifying targets in low-concentration samples^9,10^. However, the reliability of gene expression measurements is strongly affected by technical factors, such as template quality, efficiency of complementary DNA (cDNA) synthesis, primer performance, and data normalization^11,12^. The latter, when inappropriately performed, compromises the conclusions of studies^13,14^. There are many options for normalizing the quantification of gene expression, such as employing similar sample sizes for extraction, employing similar amounts of RNA for cDNA synthesis, targeting genomic DNA, and employing reference genes. The use of reference genes is the most commonly employed option because of its potential to remove most technical variations in cDNA concentrations between samples^15^.

Reference genes, also referred to as the internal control, constitutive or housekeeping genes, must not vary across experimental or biological conditions. However, several studies choose constitutive genes without appropriate validation, assuming expression stability^16^. The internal control genes classically chosen for gene expression studies in mammals are glyceraldehyde-3 phosphate dehydrogenase (*GAPDH*), beta-actin (*ACTB*), beta-2-microglobulin (*B2M*)*,* glucose-6-phosphate dehydrogenase *(G6PD)*, beta-D-glucuronidase (*GUSB*), peptidylprolyl isomerase A (*PPIA*), and hypoxanthine phosphoribosyltransferase 1 (*HPRT*) for their crucial cellular functions and constitutive nature^17,18^. Nevertheless, previous studies have questioned the stability of these controls in different conditions^17,18^ and the practice of using only one normalizer gene^16^.

Therefore, according to the Minimum Information for Publication of Quantitative Real-Time PCR Experiments guidelines, to determine gene expression, some specific prerequisites must be followed, such as the choice of a stable internal control in the experimental conditions to be compared^19^. In this study, we selected reference genes with their stability already assessed in endometriosis, endometrial cancer, and mesenchymal stem/stromal cells (MSCs) under different experimental conditions (see Supplementary Table S1). To date, no study has reported the stability of reference genes for the normalization of RT-qPCR data for MenMSCs from women with and without endometriosis. Elucidating the roles played by these cells in the etiopathogenesis of endometriosis has been the object of extensive investigation in recent years^4,20^. For this reason, this novel study may help the scientific community to choose a reliable internal control and an optimal number of reference genes in expression studies investigating MenMSCs in endometriosis.

## Results

This exploratory study evaluated 32 known reference genes for the normalization of RT-qPCR data under experimental conditions involving the two-dimensional cultivation of MenMSCs obtained from healthy women (n = 5) and women with endometriosis (n = 5). These cells were stored in a biorepository and were previously characterized as MSCs by a panel of 23 markers. The marker expressions are in agreement with the recommendations of the International Society for Cellular Therapy^21^ and the immunophenotypic profile of MenMSCs^20^ (see Supplementary Table S2). These cells have the capacity to differentiate in vitro into typical adipogenic and osteogenic mesodermal lineages (data not shown).

### Expression profile of candidate reference genes

**Figure 1:**
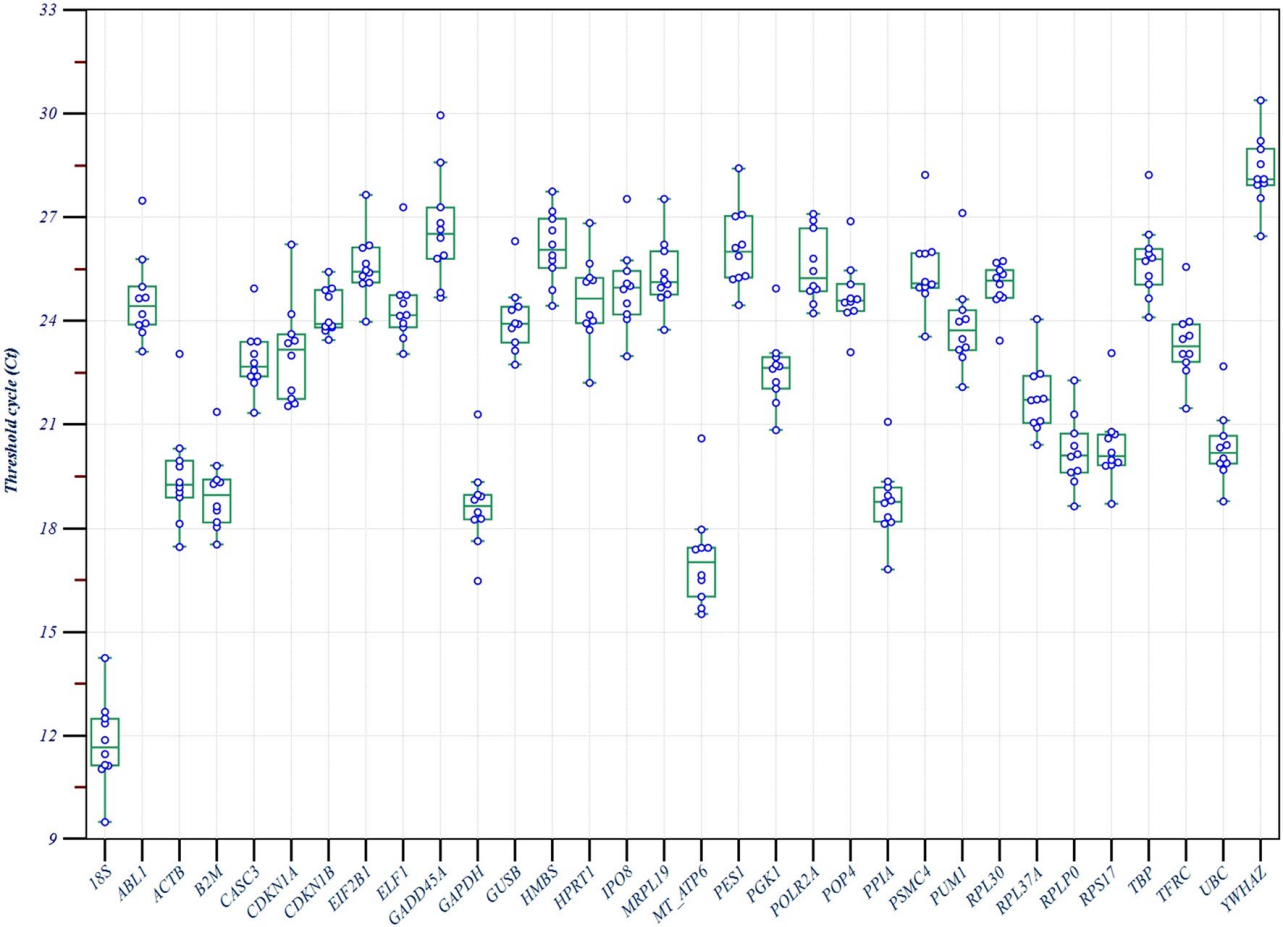
Box plots and dots of Ct values for the 32 candidate reference genes. The box represents the values from the lower (Q1—25th percentile) to the upper quartile (Q3—75th percentile). The horizontal line inside the box represents the median. Minimum [Q1 − (1.5*IQR)] and maximum [Q3 + (1.5*IQR)] values are represented by whiskers, excluding outlier values, which are displayed as separate points (blue circles outside). The sample distribution is represented by blue circles. *IQR* interquartile range. The box-plot was generated using MedCalc statistical software version 19.5.1 (MedCalc Software Ltd, Ostend, Belgium; https://www.medcalc.org; 2020).

The distribution of cycle threshold (Ct) values of the candidate reference genes is shown in Fig. 1. *18S* was the most abundantly expressed gene (Ct value min 9.5 and max 14.2), and *YWHAZ* was the least expressed (Ct min 26.4 and max 30.3). Approximately 78% of genes were in the range of 20–27. The *GADD45A* gene had the highest full range of variation (from Ct min to max), representing a large intragroup amplitude variation. The Ct values in the endometriosis group were higher (Table 1), but there were no significant differences when compared to the control group, except for the *MT-ATP6* gene (*P* = 0.03). This gene was excluded from the stability analysis because it did not meet the requirements of the NormFinder algorithm, i.e. the average expression level must be similar between groups^22^.

**Table 1:** Ct values for 32 reference genes in experimental groups.

Gene ID	Control Ct value (mean ± SD) n = 5	Endometriosis Ct value (mean ± SD) n = 5
*18S*	11.33 ± 1.17	12.25 ± 1.30
*ABL1*	23.97 ± 0.56	25.33 ± 1.42
*ACTB*	18.89 ± 1.01	20.15 ± 1.69
*B2M*	18.46 ± 0.68	19.57 ± 1.20
*CASC3*	22.31 ± 0.62	23.40 ± 0.96
*CDKN1A*	22.48 ± 0.96	23.66 ± 1.72
*CDKN1B*	23.98 ± 0.56	24.54 ± 0.69
*EIF2B1*	25.16 ± 0.67	26.03 ± 1.04
*ELF1*	23.81 ± 0.56	24.99 ± 1.34
*GADD45A*	25.93 ± 0.80	27.46 ± 1.94
*GAPDH*	18.07 ± 1.02	19.23 ± 1.24
*GUSB*	23.58 ± 0.63	24.54 ± 1.10
*HMBS*	25.86 ± 0.91	26.39 ± 1.17
*HPRT1*	24.10 ± 1.23	25.13 ± 1.20
*IPO8*	24.27 ± 0.85	25.63 ± 1.16
*MRPL19*	24.87 ± 0.64	25.84 ± 1.17
MT-ATP6*	16.26 ± 0.76	17.99 ± 1.55
*PES1*	25.60 ± 0.72	26.60 ± 1.36
*PGK1*	22.16 ± 0.88	23.00 ± 1.15
*POLR2A*	25.08 ± 0.59	26.01 ± 1.23
*POP4*	24.29 ± 0.66	25.20 ± 1.08
*PPIA*	18.38 ± 0.94	19.14 ± 1.17
*PSMC4*	24.89 ± 0.87	26.03 ± 1.31
*PUM1*	23.22 ± 0.81	24.59 ± 1.51
*RPL30*	24.75 ± 0.85	25.27 ± 0.36
*RPL37A*	21.33 ± 0.58	22.20 ± 1.26
*RPLP0*	19.77 ± 0.68	20.67 ± 1.20
*RPS17*	19.81 ± 0.71	20.92 ± 1.25
*TBP*	25.12 ± 0.77	26.37 ± 1.13
*TFRC*	22.70 ± 0.77	24.00 ± 0.95
*UBC*	19.80 ± 0.60	20.91 ± 1.12
*YWHAZ*	27.73 ± 0.79	28.93 ± 0.98

*P < 0.05. Wilcoxon rank sum test for independent samples.

### Coregulated candidate reference genes

Coregulated genes may increase the chance of being falsely classified as stably expressed by methods that use the pairwise comparison approach^23^. Robust patterns of coexpression can inform coregulated genes by a common transcription factor when the correlation between their expression is greater than 0.84^24^. To exclude any potential bias, we detected coexpressed genes with a confidence interaction score ≥ 0.7 (see Supplementary Fig. S1). Therefore, we excluded three coregulated genes (*RPL30, RPL37A,* and *RPS17*) from our list of candidate reference genes and retained the *RPLP0* gene because of its stability in experimental studies on MSCs (see Supplementary Table S1).

### Stable reference genes

To determine the stability and ranking of the candidate reference genes, we used geNorm^16^, NormFinder^22^, BestKeeper^25^, and delta-Ct^26^ algorithms available within RefFinder web-tool^27^. Next, the comprehensive final overall ranking was calculated using this web-tool based on these four algorithms. Moreover, we applied the original NormFinder (Excel add-in) to compare the ranking of the genes and the measure of the absolute stability value with the NormFinder algorithm of RefFinder, as these output data were examined for the different NormFinder applications^28^.

**Figure 2:**
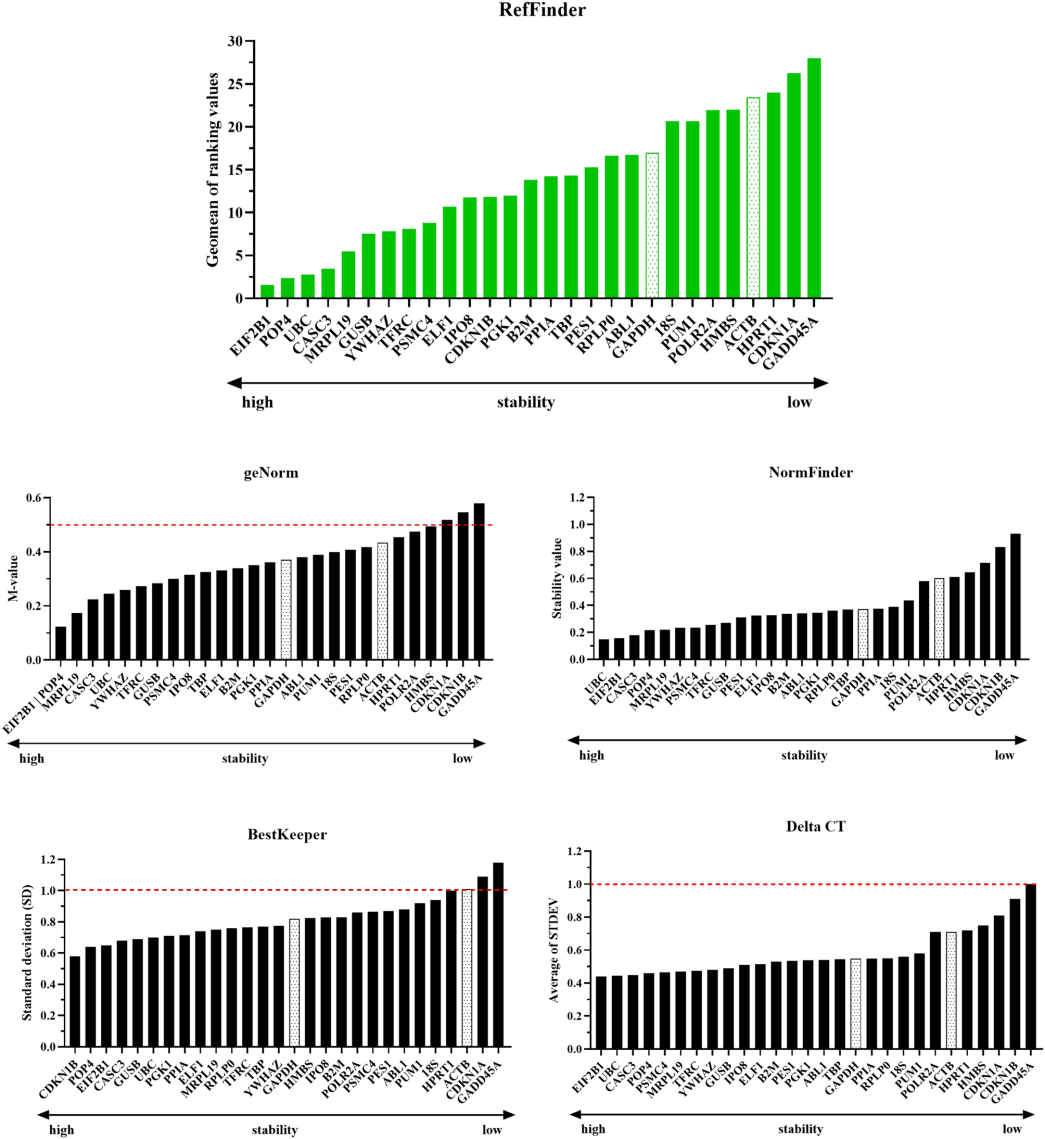
The final overall ranking of the 28 genes using RefFinder tool, and the stability of expression for the genes calculated using geNorm, NormFinder, BestKeeper, and the comparative delta-Ct (available in RefFinder). The least stable genes are on the right and the most stable genes are on the left. Highlight bars represent *GAPDH* and *ACTB* genes. The dash line indicates the cut-off value. n = 10. It was performed using GraphPad Prism version 8.0.1 for Windows (GraphPad Software, San Diego, California USA, www.graphpad.com).

According to the final overall ranking of the 28 candidate reference genes using RefFinder, the top four genes were *EIF2B1* > *POP4* > *UBC* > *CASC3* (starting from the most stable to the least stable) (Fig. 2). It was also observed that *GAPDH* and *ACTB* (more commonly used as normalizers in literature) were in the 20th and 25th positions, respectively (highlighted bars).

The results for each program were (Fig. 2): **geNorm**. This tool ranked genes based on the geNorm M value (average expression stability) and good reference genes have an M < 0.5 for homogeneous samples. In our study, 25 genes were indicated to be good reference genes because they had M < 0.5. The top four genes were *EIF2B1/POP4* > *MRPL19* > *CASC3. EIF2B1* and *POP4* were considered highly stable because they had the lowest geNorm M value; **NormFinder**. According to output data from NormFinder approachable on RefFinder, the top four genes were *UBC* > *EIF2B1* > *CASC3* > *POP4*. The most stable reference genes are those exhibiting the lowest stability value (SV), but a cut-off value is not suggested; **BestKeeper.** As stated by the BestKeeper algorithm, the *ACTB*, *CDKN1A,* and *GADD45A* genes were unstable and considered inconsistent having standard deviation (SD) > 1 (high variation). The four most stable genes were *CDKN1B* > *POP4* > *EIF2B1* > *CASC3;* **Delta-Ct method.** Based on delta-Ct comparative method, the top four genes were *EIF2B1* > *UBC* > *CASC3* > *POP4.* The stability was estimated using the SD means by pairwise comparison of two genes; SD < 1 represent stable expression. In our analyses, 27 genes are stable.

All four statistical algorithms ranked the four most stable reference genes with slight differences in their respective orders. *EIF2B1* was ranked first by the delta-Ct method and geNorm, and second by NormFinder. *POP4* and *CASC3* were classified in the top four by all statistical algorithms. However, *POP4* was ranked 1st and 2nd by geNorm and BestKeeper, respectively, whereas *CASC3* was ranked 3rd and 4th. *UBC* was ranked among the top four by two algorithms (1st by NormFinder and 2nd by delta-Ct). Interestingly, this gene was not classified among the top four genes by BestKeeper and geNorm. Despite these dissimilar ranking results, all four statistical algorithms classified *GADD45A* as the least stable gene.

The NormFinder is a mathematical model that estimates overall gene expression variation of both the intra- and intergroup and then, combines the two into a stability value^22^. The ranking of the genes in the original NormFinder (Excel Add-in) differed from the NormFinder within RefFinder when the group identifier was included. Interestingly, the greater impact of these analyses was on genes with moderate stability (18 of 28 genes). However, the classification of genes was the same between the original NormFinder (Excel Add-in) calculation considering only the intragroup variation and the RefFinder implementation, but with the measures of absolute stability being different (see Supplementary Fig. S2). In all comparisons, the five most stable genes remained ranked among the top five genes. Therefore, for the set of samples we tested, the combined inter- and intragroup analyses are unlikely to be relevant to the identification of the five most stable genes, with *EIF2B1* being ranked first by geNorm, NormFinder (Excel add-in), and delta-Ct.

### Optimal number of reference genes

**Figure 3:**
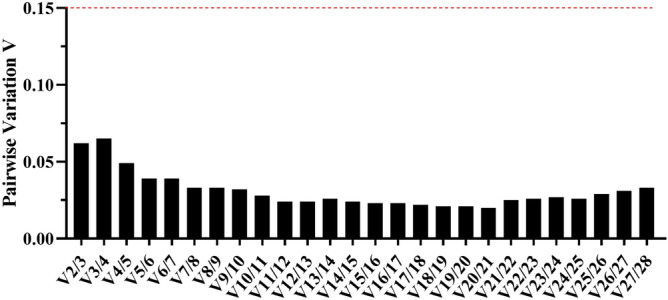
Pairwise variation measured using geNorm to determine the optimal number of reference genes for normalization. The dash line indicates the cut-off value of 0.15. It was performed using GraphPad Prism version 8.0.1 for Windows (GraphPad Software, San Diego, California USA, www.graphpad.com).

All pairwise variation values calculated using the geNorm algorithm fell below a cut-off value of 0.15 in our study (Fig. 3). Therefore, in this study, the V2/3 value indicated that two candidate genes were sufficient for suitable normalization of RT-qPCR data.

## Discussion

This report describes the first systematic stability analysis of a panel of 32 housekeeping genes evaluated using four statistical algorithms in the MenMSCs of healthy women and women with endometriosis. According to the current consensus, it is important to evaluate the stability of internal control genes and to determine the optimal number of reference genes for RT-qPCR analysis under specific experimental conditions. Inaccurate data might be generated if an inappropriate reference gene is selected^10,29,30^.

Through descriptive statistics using raw Ct values, we observed that approximately 78% of genes were in the Ct range of 20–27. The variation in Cts of the reference genes is important when choosing a good normalizer gene. In addition to stability, it is desirable that the reference genes have Ct values similar to that of the target gene of the study^31^. A suitable housekeeping gene should not have very low (Ct > 30) or very high (Ct < 15) expression^32^. The Ct values demonstrated a variable biological behavior of the 32 analyzed genes with a possible negative modulation of expression level in endometriosis. This observation is expected because several deregulated cellular events are necessary for the establishment of ectopic tissue, for example: increased cell adhesion, invasion and proliferation, decreased apoptosis, and induction of angiogenesis, that is, loss of cellular homeostasis in endometriosis^5^.

Any statistical presentation applying raw Ct values should be carefully evaluated, as Ct is an exponential and not a linear term^33^. For this reason, to increase accuracy when the stability of candidate reference genes is assessed, different statistical algorithms, such as geNorm^16^, NormFinder^22^, BestKeeper^25^, and the comparative delta Ct method^26^ are used. In our analysis, the general classification of genes had different results as per the four algorithms, except for the first four positions of the most stable genes, which were very similar. These discrepancies were probably because the algorithms employ different approaches. Therefore, we used the RefFinder integration tool to make the final ranking decision^27^. This comprehensive method has been widely used for studying different species and experimental conditions, and provides a helpful integration of tools to identify reference genes^34–38^. It is important to highlight that this integrative tool does not estimate intergroup variations via NormFinder. However, for our analysis it is unlikely to be relevant for the identification of the five most stable genes.

We applied 28 of the 32 genes previously chosen for the stability analysis as two important points must be considered. First, coregulated genes have a similar expression profile^24^. Therefore, they can be erroneously ranked at the top, as they are stably expressed by methods using the pairwise comparison approach^23^. Second, we also excluded the *MT-ATP6* gene with differential expression between groups (control and endometriosis) to comply with the NormFinder requirement^22^.

According to the RefFinder tool and the optimal number of reference genes determined using geNorm, *EIF2B1* and *POP4* were the two most stable genes for MenMSCs from women with and without endometriosis. As reported in the literature, *EIF2B1* and *POP4* have many functions. *EIF2B1* is an encoder of a five-subunit component of the protein eukaryotic initiation factor 2, specifically the alpha subunit. This protein is an initiation factor involved in protein synthesis and mediates the binding of methionyl-tRNAi to 40S ribosomes in a GTP-dependent manner^39^. *POP4* (POP4 homolog, ribonuclease P/MRP subunit) encodes RPP29, one of the protein subunits of the small nuclear ribonucleoprotein complex RNase mitochondrial RNA processing endoribonuclease involved in processing precursor rRNA and RNase P (ribonuclease P), a ubiquitous ribozyme that catalyzes 5′-maturation of tRNAs, as reviewed by Hartmann and Hartmann^40^. Because of the crucial cellular functions of these genes, they are considered to have a constitutive nature.

The eMSCs and MenMSCs exhibit properties similar to bone marrow MSCs, as reviewed by Gargett et al.^20^. In general, for MSCs, the stability of reference genes can be affected not only due to different experimental conditions but also depending on the source of their origin^41^. The same gene panel used in the present study was evaluated in MSCs obtained from different tissue sources and in varied experimental conditions^42–44^. Although there is no congruence of results regarding the most stable genes, these studies corroborate our findings regarding the instability of *GAPDH* and *ACTB*, which are used as single control genes in most high-impact journal publications^45^.

To better understand the participation of MSCs obtained from the endometrium, menstrual blood, and endometriotic lesions in the etiopathogenesis of endometriosis, high-throughput methodologies have been used^46–49^. These studies used *GAPDH, RPL19,* and *ACTB* as single reference genes for RT-qPCR data. However, the arbitrary selection of a single internal control is not appropriate^19^. Our original findings may establish a foundation for future studies investigating gene expression in eMSCs from women with and without endometriosis. We also believe this is a high-visibility study capable of directing different experimental designs, as MenMSCs are derived from a minimally invasive tissue source with fewer ethical concerns for obtaining them and with multifunctional roles in regenerative medicine^50^.

Interestingly, we found the five most stable genes (*EIF2B1, POP4, UBC, CASC3,* and *MRPL19)* were not previously measured in endometriosis expression studies. These genes were determined to be more stable than *ACTB, B2M, GAPDH, GUSB, HPRT1, PPIA, TBP,* and *YMHAZ*, which had their stability already analyzed in comparative studies between endometrial eutopic and ectopic tissue^14,51^. We strongly encourage the inclusion of these genes in future RT-qPCR experiments in the context of endometriosis.

The main limitation of this study is the small sample size. We adopted stringent case and control selection criteria, as it is known that homogeneous samples are best suited to highlight biological effects. However, our results from NormFinder analysis need to be interpreted carefully, as NormFinder requires at least 8 biological replicates per group and 5–10 candidate genes for good estimates^22^. Therefore, it is important to integrate these data with the other three statistical algorithms and evaluate a greater number of candidate genes for a reliable selection of reference genes. Another issue to be addressed is that these cells do not represent the real cellular molecular regulatory environment, as they were analyzed after cell culture, which makes it difficult to assume the same results for in vivo systems. However, in vitro studies are still the best choice for research in this investigation field.

In summary, we recommend the use of two reference genes for the normalization of RT-qPCR data in studies with MenMSCs from women with and without endometriosis. Our study suggests that *EIF2B1* and *POP4* are the most stable genes. Moreover, we recommend avoiding the use of *GAPDH* and *ACTB* in study designs similar to ours, as they were determined to be unstable.

## Methods

The MenMSCs used in this study are part of a biorepository of the Human Reproduction Section at the Department of Gynecology and Obstetrics of the Ribeirao Preto Medical School. They were collected from November 2014 to December 2016 following the ethics guidelines established by the Declaration of Helsinki. This study and the MenMSCs collection were approved by the Research Ethics Committee of the Clinical Hospital of the Ribeirao Preto Medical School (HCRP 15227/2012). Informed consent was obtained from all the participants enrolled in the study.

### Patient characteristics

Eligible patients (n = 10; five women with endometriosis and five healthy controls) were women between 18 and 40 years of age with regular menstrual cycles (intervals from 24 to 32 days ± 3 days; 2 to 7 days of duration). These women had not used any type of hormone therapy at least 3 months prior to sample collection and did not have any systemic disease, such as diabetes mellitus, or any endocrinopathy, cardiovascular disease, systemic lupus erythematosus, or other rheumatological diseases; additionally, they did not have smoking and drinking habits.

For the endometriosis group, five women at the mean age of 34.8 (SD ± 3.1) were selected, with videolaparoscopic and histological diagnosis of stage III or IV endometriosis as per the criteria defined by ARSM^52^. Only patients who had undergone surgical treatment an average of 4 years (SD ± 1.3) prior to sample collection were selected. Only patients presenting an imaging diagnosis suggestive of endometrioma prior to sample collection as an indication of active disease were enrolled in the study. For the control group, five fertile women (at least with two children) were selected; their mean age was 34.2 years (SD ± 4.0) with no history of recurrent abortion and no clinical or videolaparoscopic diagnosis of endometriosis, who had undergone videolaparoscopy for tubal ligation. Some patients’ details are reported in Table 2.

**Table 2:** Characteristics and clinical data of patients with and without endometriosis.

Control	Age (years)	BMI (kg/m2)	Menstrual cycle day	Endometriosis	Age (years)	BMI (kg/m2)	Menstrual cycle day	ASRM Classification	USG/ endometrioma	Symptoms
C1	33	23.5	4	E1	31	28.1	3	III	LO	Inf/pain
C2	39	26.4	3	E2	34	25.7	3	IV	RO/LO	Inf/pain
C3	36	24	2	E3	33	29.4	4	III	RO	Inf/pain
C4	35	25.2	3	E4	39	24.6	4	IV	LO	Inf/pain
C5	28	28.2	3	E5	37	25.5	2	III	LO	Inf
**Mean** **(SD)**	34.2 (4.0)	25.4 (1.8)	3 (0.7)	**Mean** **(SD)**	34.8 (3.1)	26.6 (2.0)	3.2 (0.8)			

*BMI* body mass index; *USG* ultrasonography; *LO* left ovary; *RO* right ovary; *Inf* Infertility.

### Sample collection and MenMSC isolation

Menstrual blood was collected in a sterile menstrual cup (Inciclo, Brazil) inserted deeply into the vagina for 3 h during the 2nd, 3rd or 4th day of the menstrual cycle. The sample was transferred to a solution containing phosphate-buffered saline plus 10 × antibiotic–antimycotic (# 15240-062, Gibco, USA) and 10% acid-citrate-dextrose solution (JP FARMA, Brazil). Next, the sample was stored at 4 °C for 4 h. Human MenMSCs were obtained according to a previously reported protocol^53^. Mononuclear cell separation was performed via density gradient centrifugation at 800 g for 30 min at 22 °C with Ficoll-Paque (#71-7167-00AG, GE Healthcare Bio-Sciences, Sweden). The interphase cells were transferred to α-minimum essential medium (# 11900-016, Gibco, USA) containing 15% fetal bovine serum (# SH30071.03, GE Healthcare—HyClone, USA), 1% penicillin/streptomycin (# 15140-122, Gibco, USA), 10 mM HEPES (# H4034, Merck, USA), and 20 mM sodium bicarbonate (# 56297, Merck, USA). Cell culture media was changed every 2–3 days until adherent cells grew to 80–90% confluence. The cells were subcultured using 0.05% trypsin–EDTA solution (#25300054, Gibco, USA). Third passage (early culture) cells were employed.

MenMSCs met the minimal criteria for multipotent MSCs as defined by the International Society for Cellular Therapy^21^. The cells were immunophenotypically characterized via a panel of 23 markers using the FACSCalibur flow cytometer (BD Biosciences, USA) and the CellQuest™ software version 4.0 (BD Biosciences, USA). The immunophenotyping panel composition is available in Supplementary Table S2. Moreover, upon meeting the characterization criteria suggested by the International Society for Cellular Therapy, the cells were differentiated into adipocytes and osteocytes following the conditions of the differentiation medium described by Musina et al^54^. We used 4 × 10^4^ cells/mL to induce differentiation and 5 × 10^3^ cells/mL for control. Cells were cultured in GREINER CELLSTAR 24-well plates (# M8812, Merck, USA) with Deckgläser cover slips (# 100013, Knittel, Germany). Cell differentiations were induced 24–48 h after the start of cultivation and were performed for 15–20 days for adipogenic differentiation and 31 days for osteogenic differentiation.

### RNA isolation and cDNA synthesis

Total RNA was extracted from MenMSCs using the AllPrep DNA/RNA/miRNA Universal Kit (#80224, Qiagen, USA) according to the manufacturer’s instructions. After the treatment of the samples with Ambion DNA-free Kit DNase Treatment and Removal (#AM1906, Invitrogen, USA), the quality of the RNA samples was evaluated with the Agilent RNA 6000 Nano Kit (# 5067-1511, Agilent, USA) using the Agilent 2100 Bioanalyzer (Agilent, USA). Only samples with RNA integrity number (RIN) ≥ 7 were included in the study. Total RNA was quantified with the Qubit RNA BR Assay Kit (#Q10210, Invitrogen, USA) using the Qubit 2.0 Fluorometer (Invitrogen, USA) (see Supplementary Fig. S3). Briefly, cDNA was synthesized with 1000 ng of total RNA using a High-Capacity RNA-to-cDNA Kit (#4387406, Applied Biosystems, USA) according to the manufacturer’s instructions. The final cDNA product was diluted 1:5 with nuclease-free water and stored at − 20 °C until use.

### RT-qPCR

The expression levels of 32 validated reference genes were assessed using the TaqMan Array Human Endogenous Control Plate, Fast 96-well (#4396840, Applied Biosystems, USA) (see Supplementary Table S3). ThermoFisher Scientific made these genes available after selection from literature studies performed on various human tissues and also because they have been shown to be expressed constitutively and with moderate abundance in most samples (see application note—Using TaqMan endogenous control)^55^. RT-qPCR amplifications were performed using a ViiA 7 Real-time PCR System (Applied Biosystems, USA). Each reaction was prepared in a total volume of 10 µL containing 5.0 µL of TaqMan Fast Advanced Master Mix (2X) (#4444557, Applied Biosystems, USA), 0.5 µL of hydrolysis probe (20X), and 4.5 µL of 1:5 dilution of the cDNA. All samples were run in triplicate on fast 96-well reaction plates. We considered replicates with the maximum difference between Ct values up to 0.3 cycles. The cycles for RT-qPCR were as follows: one cycle at 50 °C for 2 min, one cycle at 95 °C for 20 s, 40 cycles of 1 s at 95 °C and 20 s at 60 °C. Thermo Fisher Scientific guarantees that the amplification efficiency of the assays is very close to 100% and states that it is not necessary to measure the efficiency (see application note. Amplification efficiency of TaqMan Gene Expression Assays)^56^. A reverse transcriptase-minus control and no template control containing RNAse-free water instead of template mRNA were included in only one plate run. No amplification was detected confirming the absence of contamination.

### Differentially expressed and coregulated candidate reference genes

We used the STRING database (https://string-db.org/) to find coregulated genes with high confidence interaction score (≥ 0.700)^57^. To estimate differences in the expression of candidate reference genes between the control and endometriosis groups, we transformed the raw Ct to 2 ^ Ct and carried out the Wilcoxon rank sum test for independent samples using R software version 4.0.1^58^. The level of significance was defined as *P *value less than 0.05.

### Analysis of reference gene stability

Data analysis was performed with the combined set of samples (control and endometriosis groups). The stability of the candidate reference genes was estimated using the mean Ct values of the three technical replicates for each sample using the web-based comprehensive tool RefFinder^27^. This web-tool includes four different statistical algorithms:

1. The geNorm algorithm calculates the geNorm M value though a geometric averaging of all the reference genes used in the study and the mean pairwise variation of a reference gene from other genes evaluated^16^. Lower M values indicate greater stability: values below M ≤ 1.0 are recommended by geNorm. Good reference genes have an M < 0.5, whereas M values up to 1 are acceptable for heterogeneous samples^59^;
2. NormFinder classifies candidate genes according to the calculation of the SV by considering intra- and intergroup variations for candidate reference genes^22^. We used the original NormFinder (Excel Add-in), version 0.953, to compare the ranking of the genes to the NormFinder algorithm within RefFinder web-tool. The NormFinder (Excel Add-in) is available at https://moma.dk/normfinder-software;
3. BestKeeper determines the stability of candidate reference genes based on SD and coefficient variation (CV) of their raw Ct values, and any gene with SD > 1 is considered unstable^25^;
4. comparative delta Ct calculates the relative expression between the candidate and other genes within the same sample and ranks them based on the reproducibility of expression variation among the study samples^26^.

RefFinder ranks candidate reference genes by integrating the results of the four programs. This tool allocates an appropriate weight to each gene and then calculates the geometric mean of their weights to obtain an overall ranking.

### Determination of the optimal number of reference genes

The optimal number of genes required for more accurate normalization was calculated using the statistical algorithm geNorm available on the qbase Plus software version 3.2^59^. The algorithm identifies the optimal number of genes determining the V value using pairwise variation (V_n_/V_n+1,_ n = the number of candidate reference genes) between two sequential normalization factors (NF) NF_n_ and NF_n+1_ for all samples. Additionally, it recommends a cut-off value of 0.15. Values below 0.15 indicate that inclusion of an additional reference gene is not required.

## Supplementary Information

Supplementary Legends.

Supplementary Tables.

Supplementary Information 2.

Supplementary Information 3.

Supplementary Information 4.
