# Supplementary material for: Identification of suitable reference genes for mesenchymal stem cells from menstrual blood of women with endometriosis
Source: Sci Rep. 2021 Mar 8;11:5422. doi: 10.1038/s41598-021-84884-5 (PMC7970877; doi:10.1038/s41598-021-84884-5)
Supplement: Supplementary file 1 — Supplementary Legends. [file 41598_2021_84884_MOESM1_ESM.doc]

**ADDITIONAL INFORMATION**

**Supplementary information accompanies this paper.**

**Supplementary Table S1**. Reference gene with stability assessed in studies of mesenchymal stem cells (MSCs), endometriosis and endometrial cancer.

**Supplementary Table S2**. TaqMan human endogenous control genes used in the study, providing gene symbol, name, primary function, and assay number.

**Supplementary Table S3.** Markers used for the immunophenotypic characterization of MenMSCs using flow cytometry.

**Supplementary Figure S1.** A protein-based coexpression analysis using the STRING database for the candidate reference genes. The black lines represent coexpression with a high confidence interaction score (≥0.7). The image was generated using STRING software version 11 available on the web (<https://string-db.org/>).

**Supplementary Figure S2.** Ranking of the genes by NormFinder available in RefFinder and the original NormFinder (Excel add-in) with and without group identifier. Red bars represent the genes that had their position changed in the RefFinder. It was performed using GraphPad Prism version 8.0.1 for Windows (GraphPad Software, San Diego, California USA, [www.graphpad.com](http://www.graphpad.com/)).

**Supplementary Figure S3.** The electropherograms of the samples were obtained by applying the Agilent 2100 Bioanalyzer and RNA 600 Nano kit. RNA Integrity Number (RIN) was performed using Agilent 2100 Expert B.02.07.SI532 software. C: control; E: endometriosis. The numbers and letters A or B represent the sample code in the biorepository. [ ] = concentration of RNA obtained by Qubit 2.0 Fluorometer.
