# Supplementary material for: Identification of suitable reference genes for mesenchymal stem cells from menstrual blood of women with endometriosis
Source: Sci Rep. 2021 Mar 8;11:5422. doi: 10.1038/s41598-021-84884-5 (PMC7970877; doi:10.1038/s41598-021-84884-5)
Supplement: Supplementary file 2 — Supplementary Tables. [file 41598_2021_84884_MOESM2_ESM.pdf]

Title: Identification of suitable reference genes for mesenchymal stem cells from menstrual blood of women with endometriosis

Authors list

Victoria S Zucherato, Leticia B C Penariol, Lilian E C M Silva, Cristiana C Padovan, Omero B Poli-Neto, Julio C Rosa-e-Silva, Rui A Ferriani, Juliana Meola.

Supplementary Table S1. Reference gene with stability assessed in studies of mesenchymal stem cells (MSCs), endometriosis and endometrial.

Study	Type of cells and tissues	Tools	Analyzed Genes	Most Stable Genes	Reference
Studies of mesenchymal stem cells under different culture conditions	MSCs from: adipose tissue (n=4); Wharton's Jelly (n=4); Bone Marrow (n=4); Skin (n=4)	geNorm, NormFinder and BestKeeper	N= 5 (***HPRT1, ACTB, GAPDH, B2M*** and *RPL13A*)	*RPL13A* (adipose tissue and Wharton's Jelly) and *HPRT1* (bone marrow and skin)	Amable et al.[1]
	MSC from bone marrow (n=6) with and without osteogenic induced	geNorm	N=32 **(the same panel of our study)**	*GADD45A*, *PUM1* and *RPLP0*	Jacobi et al.[2]
	MSC from bone marrow (n=8) and stromal cells from adipose tissue (n=8)	geNorm and NormFinder	N= 9 (***18S, ACTB, GAPDH, GUSB, PPIA***, ***TBP, YWHAZ,*** *EF1A and RPL13*)	*TBP* is common reference gene and two-combination *TBP* and *YWHAZ*	Tratwal et al.[3]
	MSCs from 4 different tissue (adipose tissue, bone marrow, umbilical cord and placenta)	geNorm, NormFinder and BestKeeper	N= 8 (***18S, ACTB, B2M, GAPDH, HPRT1, PPIA***, ***TBP*** *and RPL13A)*	*B2M* and *PPIA*	Li et al.[4]
	MSCs from bone marrow (n=6) in 2D compared to 3D cultivation	geNorm and NormFinder	N=32 **(the same panel of our study)**	*TBP*, *TFRC* and *HPRT1*	Rauh et al.[5]
	MSC form bone marrow (n=5) and fetal tissue (n=5)	geNorm, NormFinder and BestKeeper	N= 10 (***18S, ACTB, B2M, HPRT1, GAPDH, TBP, PPIA, RPLP0, PGK1*** and *RPL13A*)	*RPL13A*, *B2M* and *PPIA*	Li et al.[6]
	MSCs from bone marrow of 4 controls and patients with avascular necrosis of the femoral head (ANFH) divided in 4 groups: alcohol (n=5), corticosteroid (n=5), trauma (n=5) and idiopathic (n=5)	geNorm, NormFinder and BestKeeper	N= 12 (***18S, GAPDH, B2M, ACTB, TBP, GUSB, HPRT1***, ***PPIA, PUM1,*** *ALAS1, PBGD and RPL29*)	*HPRT1* and *PIA*	Wang et al.[7]
	MSCs from adipose tissue (n=7) in proliferating or senescent conditions	geNorm, NormFinder and BestKeeper	N=16 (***GAPDH, ACTB, TBP, B2M, GUSB, RPLP0, YWHAZ, HPRT1, PGK1,***	*PPIA*	Su et al.[8]

		TFRC, UBC, PPIA, *RPS18, EEF1A1, ATP5F1 and RPL13A,*)		
MSCs from adipose tissue (n=5)	RefFinder	N= 12 (***ACTB, B2M, GAPDH***, ***YWHAZ, TBP, UBC, GUSB***, ***RPLP0, HPRT1, PPIA,*** *RPS18 and RPL13A*)	*RPL13A* and *RPS18*	Palombella et al.[9]
MSCs from adipose tissue and bone marrow treated (n=6) and untreated (n=6)	geNorm	N= 8 (***TBP, ACTB***, ***GAPDH, RPLP0,*** *SDHA, POLR2E, RPL13A and RNA28S4*)	*TBP, GAPDH, RPLP0* and *POLR2* (all cell groups); *TBP, POLR2, GAPDH* and *RPLP13A* (chondrocytes); *GAPDH, RPLP0, TBP* and *SDHA* (treated chondrocytes)	He et al.[10]
MSCs from 1 bone marrow and 1 umbilical cord were cultivated with different fetal bovine serum (n=7).	geNorm and Normfinder	N=12 (***ACTB, B2M, GAPDH, GUSB, PPIA, RPLP0, 18S, TBP, UBC, YWHAZ,*** *EF1A and RPLI3A*)	*YWHAZ* and *UBC* (bone marrow); *RPLP0* and *EF1A* (umbilical cord); and *EF1A* and *TBP* for both MSCs.	Banfi et al.[11]
MSC from different tissues and cell lines and cultivated in 2D and 3D condition (n=43).	RefFinder	N=13 (***ACTB, B2M, GAPDH, GUSB, HPRT1, PPIA, PUM1***, ***RPLP0, TBP, TRFC, YWHAZ,*** *HMBS and RPL13A*)	*TBP*	Brinkhof et al.[12]
MSCs from bone marrow (n=3) was used for validate RNAseq data.	geNorm, NormFinder and delta-Ct	N= 9 (***18S, ACTB, B2M, GAPDH, HPRT1, PGK1***, *RPL13A*, ***TBP, UBC***)	*HPRT1, PGK1* and *TBP*	Bella and Stoddart [13]
MSCs from bone marrow (n=5) and umbilical cord (n=5) were compared in long-term expanded culture	geNorm and NormFinder	N= 10 (***18S, HPRT1, B2M, RPLP0, PPIA, ACTB, YWHAZ, GAPDH, TBP and*** *EEF1A1*)	*PPIA, HPRT1* and *YWHAZ*	Jeon et al. [14]
MSCs from Wharton's Jelly (n=1) after lentiviral transduction and differentiation conditions	delta-Ct	N=32 **(the same panel of our study)**	*RPS17* and *18S*	Borkowska et al.[15]
MSCs from adipose tissues obtained in American Type Culture Collection (unspecified sample size) to compare proliferation and tri-lineage differentiation conditions.	delta-Ct and NormFinder	N=13 (***RPLP0, PPIA, B2M, HPRT1, TBP, GUSB***, ***YWHAZ, UBC, 18S, GAPDH, ACTB,*** *RPL13A and EF1A*)	*EF1A, RPLP0* and *RPL13A*	Ayanoglu et al.[16]

Endometriosis studies	Control endometrium (n=9), endometriotic lesions (n=9) and endometrium of women with endometriosis (eutopic) (n=9)	geNorm and NormFinder	N= 7 (***GAPDH, HPRT1, TBP, YWHAZ***, *HMBS, RPL13A and SDHA*)	*TBP, HMBS and YWHAZ* (lesions and eutopic endometrium); *GAPDH* and *TBP* (eutopic and control endometrium)	Vestergaard et al.[17]
	Endometriotic lesions (n=10) and eutopic endometrium (n=13)	geNorm, NormFinder and BestKeeper	N= 7 (***ACTB, B2M, GUSB, HPRT, PPIA,*** *G6PD and GAPD*)	*PPIA, GAPD* and *B2M*	Andrusiewicz et al.[18]
Endometrial Cancer	endometrial carcinoma (n=100) and normal endometrium (n=29)	geNorm and NormFinder	N= 10 (***GAPDH, B2M, ACTB, POLR2A, UBC, PPIA, HPRT1, GUSB, TBP*** and *H3F3A*)	*PPIA and HPRT1*	Romani et al.[19]
	endometrial carcinoma (n=15, was divided into 5 groups with 3 samples according to the grade of the tumor) and normal endometrium (n=9)	geNorm, NormFinder and BesteKeeper	N=32 **(the same panel of our study)**	*MRPL19, IPO8* and *PPIA*	Ayakannu et al.[20]

Note: bold genes were included in our study

REFERENCES

1. Amable, P. R., Teixeira, M. V. T., Carias, R. B. V., Granjeiro, J. M. & Borojevic, R. Identification of appropriate reference genes for human mesenchymal cells during expansion and differentiation. *PLoS One* **8**, e73792 (2013).
2. Jacobi, A. *et al.* Comparative analysis of reference gene stability in human mesenchymal stromal cells during osteogenic differentiation. *Biotechnol. Prog.* **29**, 1034–1042 (2013).
3. Tratwal, J., Follin, B., Ekblond, A., Kastrup, J. & Haack-Sørensen, M. Identification of a common reference gene pair for qPCR in human mesenchymal stromal cells from different tissue sources treated with VEGF. *BMC Mol. Biol.* **15**, 1–11 (2014).
4. Li, X., Yang, Q., Bai, J., Xuan, Y. & Wang, Y. Identification of appropriate reference genes for human mesenchymal stem cell analysis by quantitative real-time PCR. *Biotechnol. Lett.* **37**, 67–73 (2015).
5. Rauh, J., Jacobi, A. & Stiehler, M. Identification of stable reference genes for gene expression analysis of three-dimensional cultivated human bone marrow-derived mesenchymal stromal cells for bone tissue engineering. *Tissue Eng. - Part C Methods* **21**, 192–206 (2015).
6. Li, X. *et al.* Identification of optimal reference genes for quantitative PCR studies on human mesenchymal stem cells. *Mol. Med. Rep.* **11**, 1304–1311 (2015).
7. Wang, X. N. *et al.* Evaluation of the stability of reference genes in bone mesenchymal stem cells from patients with avascular necrosis of the femoral head. *Genet. Mol. Res.* **15**, 2430–2437 (2016).
8. Su, X., Yao, X., Sun, Z., Han, Q. & Zhao, R. C. Optimization of reference genes for normalization of reverse transcription quantitative real-time polymerase chain reaction results in senescence study of mesenchymal stem cells. *Stem Cells Dev.* **25**, 1355–1365 (2016).
9. Palombella, S. *et al.* Identification of reference genes for qPCR analysis during hASC long culture maintenance. *PLoS One* **12**, e0170918 (2017).
10. He, T., Huang, Y., Chak, J. C. & Klar, R. M. Recommendations for improving accuracy of gene expression data in bone and cartilage tissue engineering. *Sci. Rep.* **8**, 1–13 (2018).
11. Banfi, F., Colombini, A., Perucca Orfei, C., Parazzi, V. & Ragni, E. Validation of reference and identity-defining genes in human mesenchymal stem cells cultured under unrelated fetal bovine serum batches for basic science and clinical application. *Stem Cell*

Rev. Reports **14**, 837–846 (2018).
12. Brinkhof, B. *et al.* Improving characterisation of human Multipotent Stromal Cells cultured in 2D and 3D: Design and evaluation of primer sets for accurate gene expression normalisation. *PLoS One* **13**, e0209772 (2018).
13. Bella, E. Della & Stoddart, M. J. Cell detachment rapidly induces changes in noncoding RNA expression in human mesenchymal stromal cells. *Biotechniques* **67**, 286–293 (2019).
14. Jeon, R. H. *et al.* PPIA, HPRT1, and YWHAZ Genes Are Suitable for Normalization of mRNA Expression in Long-Term Expanded Human Mesenchymal Stem Cells. *Biomed Res. Int.* **21**, 1–11 (2019).
15. Borkowska, P., Zielińska, A., Paul-Samojedny, M., Stojko, R. & Kowalski, J. Evaluation of reference genes for quantitative real-time PCR in Wharton's Jelly-derived mesenchymal stem cells after lentiviral transduction and differentiation. *Mol. Biol. Rep.* **47**, 1107–1115 (2020).
16. Ayanoğlu, F. B., Elçin, A. E. & Elçin, Y. M. Evaluation of the stability of standard reference genes of adipose-derived mesenchymal stem cells during in vitro proliferation and differentiation. *Mol. Biol. Rep.* **47**, 2109–2122 (2020).
17. Vestergaard, A. L., Knudsen, U. B., Munk, T., Rosbach, H. & Martensen, P. M. Transcriptional expression of type-I interferon response genes and stability of housekeeping genes in the human endometrium and endometriosis. *Mol. Hum. Reprod.* **17**, 243–254 (2011).
18. Andrusiewicz, M., Słowikowski, B., Skibińska, I., Wołuń-Cholewa, M. & Dera-Szymanowska, A. Selection of reliable reference genes in eutopic and ectopic endometrium for quantitative expression studies. *Biomed. Pharmacother.* **78**, 66–73 (2016).
19. Romani, C. *et al.* Identification of optimal reference genes for gene expression normalization in a wide cohort of endometrioid endometrial carcinoma tissues. *PLoS One* **9**, e113781 (2014).
20. Ayakannu, T. *et al.* Validation of endogenous control reference genes for normalizing gene expression studies in endometrial carcinoma. *Mol. Hum. Reprod.* **21**, 723–735 (2015).

Supplementary Table S2. Markers used for the immunophenotypic characterization of MenMSCs by flow cytometry

Marker	Catalogue Number	Supplier	Marker expression. MD±SD	Classification of expressions
CD45	340040	BD Simultest	0.19 ± 0.30	negative (≤2%)
CD14	555397	BD Pharmingen	0.24 ± 0.40	negative (≤2%)
CD34	340430	BD Biosciences	0.19 ± 0.28	negative (≤2%)
CD31	555445	BD Biosciences	0.17 ± 0.31	negative (≤2%)
CD326	324204	BioLegend	0.51 ± 0.78	negative (≤2%)
CD106	551146	BD Pharmingen	0.11 ± 0.16	negative (≤2%)
HLA-DR	555813	BD Pharmingen	0.08 ± 0.12	negative (≤2%)
STRO-1	MAB1038	R&D Systems	0.87 ± 0.78	negative (≤2%)
CD51/CD61	555505	BD Pharmingen	7.37 ± 4.92	low (up to 20%)
CD146	560846	BD Pharmingen	29.66 ± 14.50	medium (up to 60%)
SUSD2	130-106-326	MACS	40.67 ± 16.69	medium (up to 60%)
CD140b	558821	BD Pharmingen	48.07 ± 17.45	medium (up to 60%)
CD166	559263	BD Pharmingen	63.03 ± 13.74	medium (up to 60%)
HLA-ABC	555552	BD Pharmingen	73.31 ± 12.70	high (up to 100%)
CD44	555478	BD Pharmingen	72.13 ± 12.57	high (up to 100%)
CD49e	555617	BD Pharmingen	80.41 ± 8.20	high (up to 100%)
CD54	555511	BD Pharmingen	84.50 ± 5.96	high (up to 100%)
CD13	557454	BD Pharmingen	86.46 ± 7.35	high (up to 100%)
CD105	560819	BD Pharmingen	75.25 ± 12.52	high (up to 100%)
CD73	550257	BD Pharmingen	84.94 ± 5.89	high (up to 100%)
CD90	555596	BD Pharmingen	93.75 ± 2.24	high (up to 100%)
CD29	559883	BD Pharmingen	94.28 ± 2.35	high (up to 100%)
Control γ1/2a	340041	BD Simultest	--------	negative isotope control

Supplementary Table S3. TaqMan human endogenous control genes used in the study, providing gene symbol, name, primary function and assay number

Gene ID	Gene Name	Function by Gene Ontology	Applied Biosystems Assay ID
18S	Eukariotic 18S ribosomal RNA	Translation	Hs99999901_s1
ABL1	V-abl Abelson murine leukaemia viral oncogene homolog 1	Protein tyrosine kinase activity	Hs00245445_m1
ACTB	Actin, beta	Structural constituent of cytoskeleton	Hs99999903_s1
B2M	Beta-2-microglobulin	Protein binding	Hs99999907_m1
CASC3	Cancer susceptibility candidate 3	RNA binding, exon junction complex	Hs00201226_m1
CDKN1A	Cyclin-dependent kinase Inhibitor 1A (p21, Cip1)	Regulator of cell cycle	Hs00355782_m1
CDKN1B	Cyclin-dependent kinase Inhibitor 1B (p27, Kip1)	Regulator of cell cycle	Hs00153277_m1
EIF2B1	Eukaryotic translation initiation factor 2B, subunit 1 alpha, 26 kDa	Translational initiation	Hs00426752_m1
ELF1	E74-like factor 1 (ets domain transcription factor)	Positive regulation of transcription	Hs00152844_m1
GADD45A	Growth arrest and DNA-damage-induced, alpha	Signal transduction in response to DNA damage	Hs00169255_m1
GAPDH	Glyceraldehyde-3-phosphate dehydrogenase	Glycolysis, regulation of translation	Hs99999905_m1
GUSB	Glucuronidase, beta	Carbohydrate metabolic process	Hs99999908_m1
HMBS	Hydroxymethylbilane synthase	Heme biosynthetic process	Hs00609297_m1
HPRT1	Hypoxanthine phosphoribosyl transferase	Generation of purine nucleotides	Hs99999909_m1
IPO8	Importin 8	Intracellular protein transport	Hs00183533_m1
MRPL19	Mitochondrial ribosomal protein L19	Translation	Hs00608519_m1
MT-ATP6	Mitochondrially encoded ATP synthase 6	ATP synthase complex	Hs02596862_g1
PES1	Pescadillo homolog 1, containing BRCT domain (zebra fish)	Cell proliferation, rRNA processing	Hs00362795_g1
PGK1	Phosphoglycerate kinase	Glycolysis, gluconeogenesis	Hs99999906_m1
POLR2A	Polymerase (RNA) II (DNA-directed) polypeptide A	Transcription initiation	Hs00172178_m1
POP4	Processing of precursor 4, ribonuclease P/MRP subunit (S. cerevisiae)	tRNA processing, mRNA cleavage	Hs00198357_m1
PPIA	Peptidylpropyl isomerase A (cyclophilin A)	Protein folding	Hs99999904_m1
PSMC4	Proteasome (prosome, Macropain) 26S subunit, ATPase, 4	ATP binding, proteolysis	Hs00197826_m1
PUM1	Pumilio homolog 1(Drosophila)	Regulation of translation	Hs00206469_m1
RPL30	Ribosomal protein L30	Translation	Hs00265497_m1
RPL37A	Ribosomal protein L37a	Translation	Hs01102345_m1
RPLP0	Ribosomal protein, large, P0	Translation	Hs99999902_m1
RPS17	Ribosomal protein S17	Structural constituent of ribosome	Hs00734303_g1
TBP	TATA box-binding protein	Transcription factor binding	Hs99999910_m1
TFRC	Transferrin receptor (P90, CD71)	Transferrin transport	Hs99999911_m1
UBC	Ubiquitin C	Protease binding, endosomal transport	Hs00824723_m1
YWHAZ	Tyrosine 3-monooxygenase/tryptophan 5-monooxygenase activation protein, zeta polypeptide	Signal transduction	Hs00237047_m1
