# Supplementary figures and images for: Identification of suitable reference genes for mesenchymal stem cells from menstrual blood of women with endometriosis
Source: Sci Rep. 2021 Mar 8;11:5422. doi: 10.1038/s41598-021-84884-5 (PMC7970877; doi:10.1038/s41598-021-84884-5)

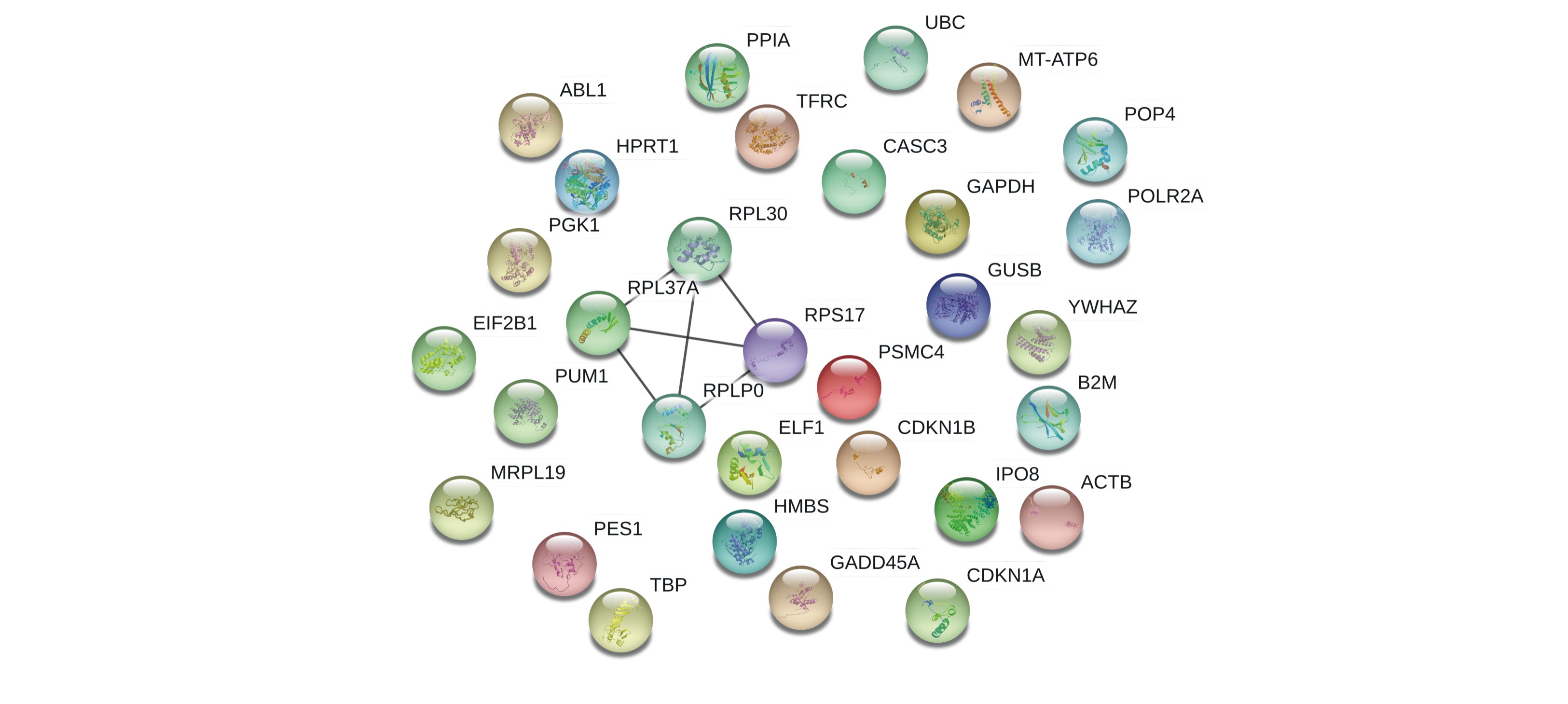

Supplement: Supplementary file 3 — Supplementary Information 2. [file 41598_2021_84884_MOESM3_ESM.jpg]

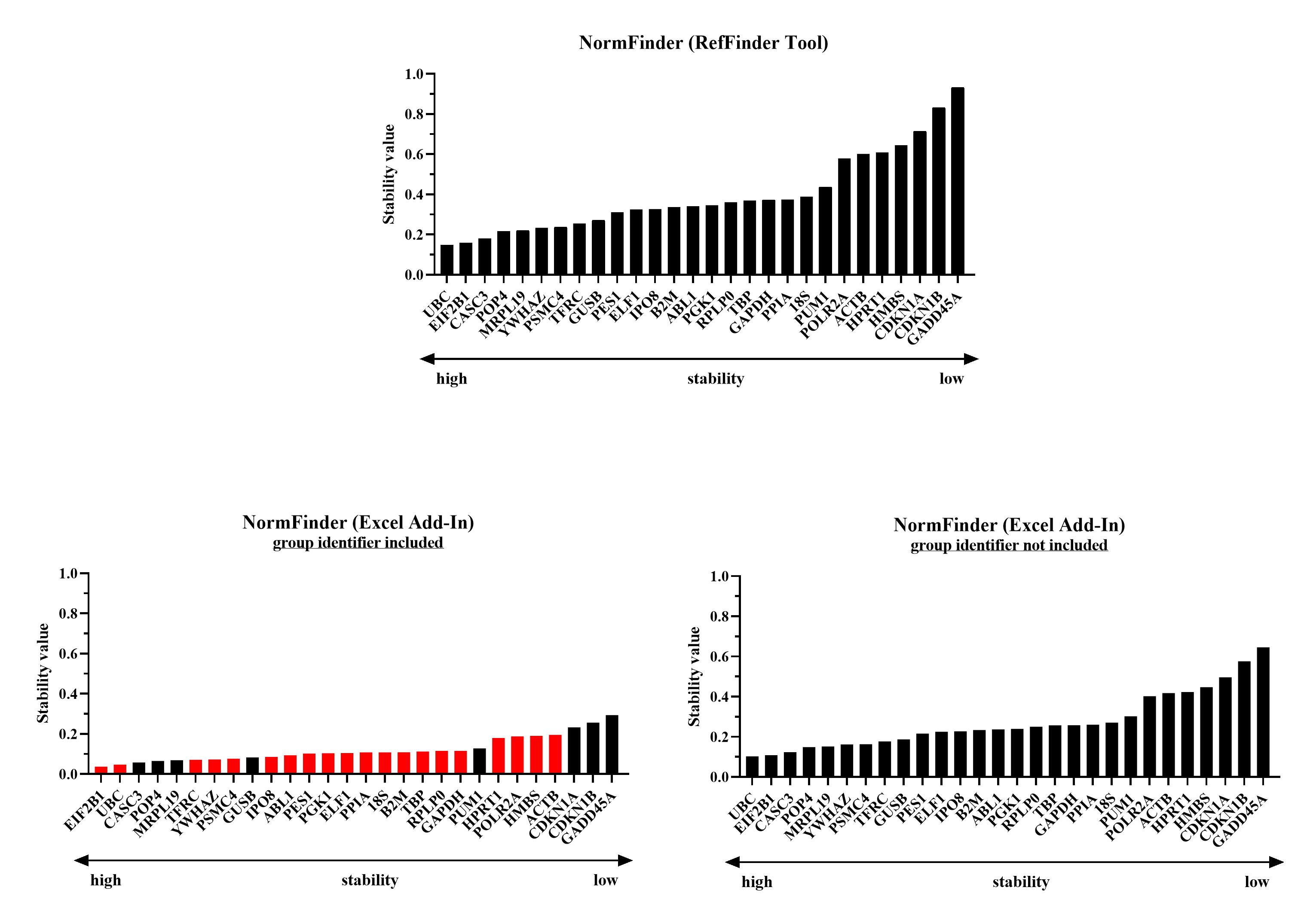

Supplement: Supplementary file 4 — Supplementary Information 3. [file 41598_2021_84884_MOESM4_ESM.jpg]

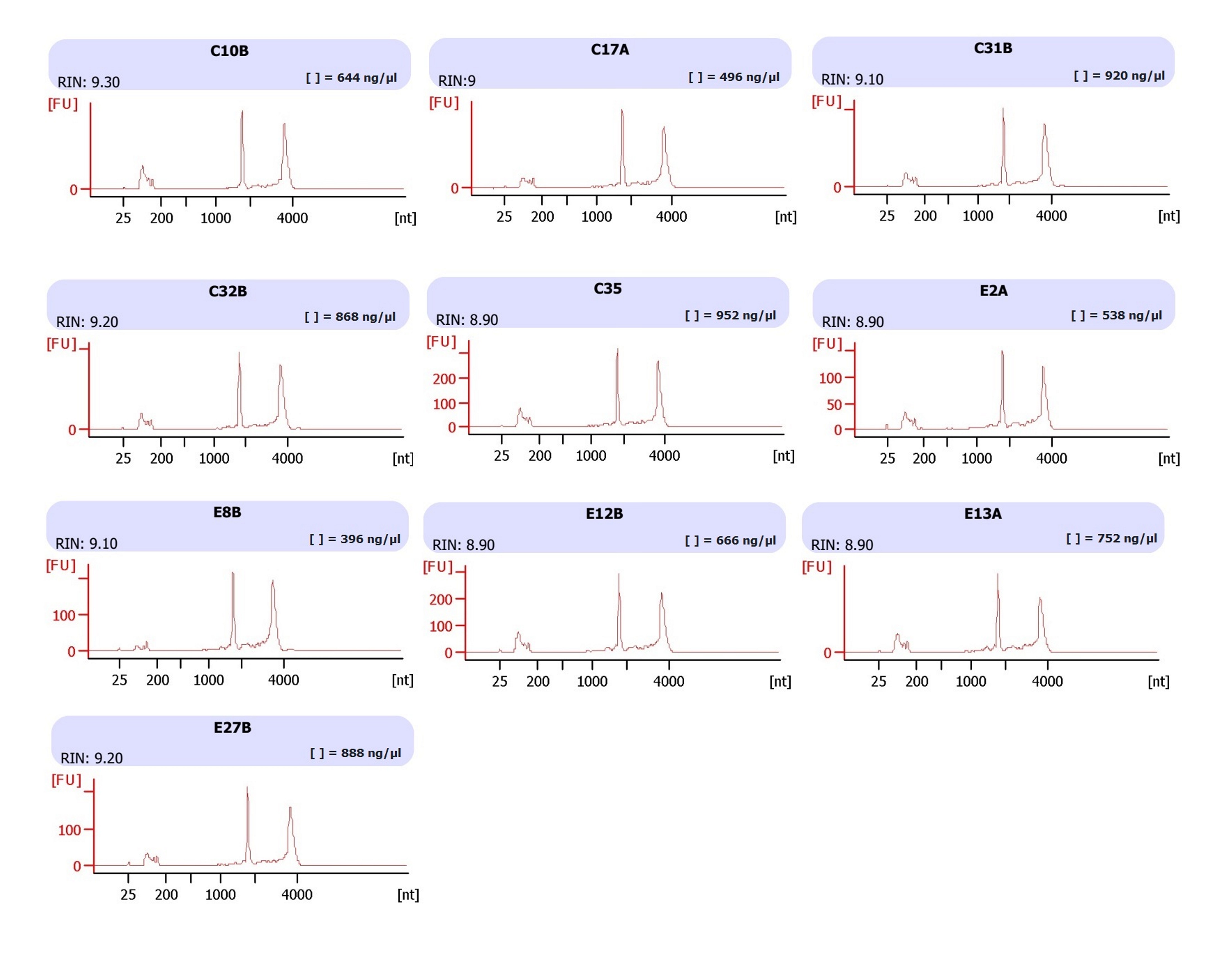

Supplement: Supplementary file 5 — Supplementary Information 4. [file 41598_2021_84884_MOESM5_ESM.jpg]
